# Evidence-based medicine

**DOI:** 10.4103/0019-5413.30518

**Published:** 2007

**Authors:** Dheeraj Shah, HPS Sachdev

**Affiliations:** Department of Pediatrics, University College of Medical Sciences and Guru Tegh Bahadur Hospital, Dilshad Garden, Delhi - 110 095; * Pediatrics and Clinical Epidemiology, Sita Ram Bhartia Institute of Science and Research, B-16 Qutab Institutional Area, New Delhi - 110 016, India

**Keywords:** Evidence based medicine, practice, research

## Abstract

Evidence based medicine is the practice of solving the clinical problems in one's practice by judicious and systematic use of the medical literature. This includes framing questions rightly and searching the right kind of literature. Thereafter, the available evidence needs to be evaluated for the validity, strength and effect size. Finally, the results are examined for applicability to the current problem which requires a detailed knowledge of the clinical setting, patient profile and the issues related to cost and harm. The present communication deals with these issues in a step-wise manner in order to stimulate readers to practise this important art.

Evidence-based medicine (EBM) means using the medical literature as a tool to solve the individual patient's or society's diagnostic, prognostic or therapeutic problems. The fact that practice should be based on evidence presented in the medical journals is widely recognized but less commonly followed. Important reasons for this include inaccessibility of good quality medical literature, paucity of time to evaluate the sea of literature and lack of determination and competence in assessing its relevance or validity to a specific patient.

One goal of EBM is to be aware of the evidence on which one's practice is based, the soundness of the evidence and the strength of the inference the evidence permits. This strategy requires a clear delineation of the relevant question(s); a thorough search of the literature relating to the questions; a critical appraisal of the evidence and its applicability to the clinical situation; and a balanced application of the conclusions to the clinical problem. The objective is to make efficient use of the published literature to help with patient care. The present communication attempts to present the basics of EBM in a simple, practical and step-wise manner and is not intended to serve as a treatise on the subject.

## STEP 1: FRAMING THE QUESTION

The evidence-based process of resolving a clinical question will be fruitful only if the problem is formulated appropriately. Here, questions raised in caring for patients are defined and then the literature is consulted to resolve these questions. Dissecting the question into its component parts to facilitate finding the best evidence is a fundamental EBM skill. Most questions can be divided into three parts:

1. The population - the relevant patients/subjects.
2. Intervention or exposure - the management strategies we are interested in comparing or the potentially harmful exposure about which we are concerned.
3. Comparison group - the alternative option which could be no intervention or some other conventional management strategy.
4. Outcome - the patient-relevant consequences or the exposure in which we are interested.

Constructing a searchable question that allows you to use the medical literature to generate an answer requires an in-depth understanding of the clinical issues involved in patient management. It requires problem-solving and analytical skills besides basic clinical knowledge about the condition. The awareness of cost and psychosocial issues is also important.

### Example

A-45 year-old multiparous women who is nondiabetic and nonhypertensive presents with low backache for duration of three months. The backache intermittently becomes severe requiring frequent analgesics. The physical and neurological examination is entirely normal. X-ray of the spine is normal.

### Initial question

Should we undertake magnetic resonance imaging (MRI) of the spine before deciding management for this woman?

### Digging deeper

The key features of this patient are her middle age, moderately severe backache, normal physical and neurological examination and a normal initial radiograph. Alternative investigational strategies address issues of the patient having any specific vertebral/spinal pathology and proceeding according to the result of this investigation.

What outcomes are we trying to influence in our choice of investigation? We would like her to be relieved of pain and disability. The reason we wish to detect vertebral/spinal lesion if they are present is that if a resectional surgery or any other specific management may benefit the patient. Thus, the primary outcome of interest is alteration of routine management (e.g., physiotherapy, analgesics etc.)

### Improved (searchable question)

A searchable question would specify the relevant patient population, the management strategy or exposure and the patient-relevant consequences of that exposure as follows:

Patients: Middle-aged women with chronic low backache

Intervention: MRI for management

Outcome:Alteration of management or surgical need.

Bearing the structure of the question in mind-patient, intervention or exposure and outcome-is extremely helpful in arriving at an answerable question. Once the question is posed, the next step in the process is translating the question into an effective search strategy.

## STEP 2: SEARCHING THE LITERATURE

After the question dealing with a patient's management is clearly defined, you need to search the literature. There are four fundamental types of clinical questions: those involving therapy (effect of different treatments on patient beneficial outcome), harm (potentially harmful effects on patient function), diagnosis (strength of a test to differentiate between those with or without a target condition) and prognosis. To answer questions about a therapeutic issue, we identify randomized controlled trials (RCTs) in which a process analogous to flipping a coin determines participants' receipt of an experimental treatment or a control or standard treatment and subjects are followed up forwardly for the outcome of interest. Ideally RCTs should also be consulted to address the issues of harm. However, for many potentially harmful exposures, randomly allocating patients is neither practical nor ethical. For these exposures, the best way is to identify observational studies in which personal choice determines whether people are exposed or not exposed. For diagnostic purposes, studies evaluate the test by comparing its performance with a gold standard or reference standard in diagnosing or excluding a particular condition. For prognosis, investigators identify patients who belong to a particular group (such as patients undergoing surgery or patients with cancer) with or without factors that may modify the prognosis (such as obesity or comorbidity) and follow them for the target outcome such as postsurgical complications or survival.

The information could be in the form of textbooks, individual study/studies, the systematic review of all the available studies, a synopsis of individual studies and systems of information. Most of these resources are now electronically available for quickly finding out the answers to the clinical questions. To find answers to general background medical questions (such as physiology, mechanism of action of an intervention or diagnostic approach to a clinical condition), referring to a textbook that is well referenced and updated frequently is likely to be faster and more rewarding. For questions dealing with specific patient's problem such as risk and benefits of a particular treatment (foreground questions), the most efficient approach is to begin with a prefiltered evidence-based resource [Table 1] in which someone has already done the exercise of reviewing the literature and choosing only the methodologically strongest studies. Looking for a systematic review article on your topic addresses a targeted clinical question using strategies that decrease the likelihood of bias. Framing the searchable question provides you with the best choice of keywords for search. Carefully modifying your search strategy by limiting the search to desired sections such as diagnostics or therapeutics would help to deal with the problem of ‘too much material’. If a search of these resources does not provide a satisfactory answer to a focused clinical question, it is the time to turn to MEDLINE. It is an attractive database for finding information because of its comprehensiveness and free accessibility. However, a thorough knowledge of the structure of database indexing, use of medical subject headings (MeSH) and combining various search results is essential for performing an effective search. Detailed information on searching MEDLINE and other reference databases is available elsewhere.^1^–^4^

**Table 1:** Online evidence-based resources

Resource	Internet address	Comments
Cochrane library	http://www.cochrane.org (requires subscription for full-text)	Largest collection of systematic reviews and bibliographic details of randomized controlled trials of healthcare interventions
Turning research into practice (TRIP)	http://www.tripdatabase.com (Free access for a limited number of times)	It searches over 55 websites of high-quality ‘evidence-based’ health information and is updated monthly
Up to date	http://www.uptodate.com (requires subscription)	Regularly updated and heavily referenced with links to full-text of original articles
Best evidence	http://www.acponline.org/catalog/electronic/best_evidence.htm (requires subscription)	Includes original studies and systematic reviews that are both methodologically sound and clinically relevant, especially for the more common diseases and conditions
Clinical practice guidelines	http://www.guidelines.gov	Provides evidence-based guidelines for common conditions
	http://www.cma.ca/cpgs (free)	
Evidence-based orthopedic trauma section in the Journal of Orthopedic Trauma	http://www.jorthotrauma.com/pt/re/jorthotrauma (requires subscription)	Provides evidence-based guidelines for managing orthopedic trauma
Evidence-based medicine section in the Journal of Bone and Joint Surgery	http://www.ejbjs.Org/cgi/content/full/82/6/759 (requires subscription)	The evidence-based section in this reputed journal details orthopedic-specific evidence-based guidelines

## STEP 3: EVALUATING THE EVIDENCE AND CLINICAL APPLICABILITY

Clinicians' most important questions involve choosing the best management strategy for their patients. For example, what are the benefits of prescribing bisphosphonates in the treatment of senile osteoporosis or mandating dietary change to influence body weight or conservative versus surgical approach for disc prolapse? The adverse impact of such treatments if any in the short and long term also needs to be examined. For each of these questions, there is a true answer which lies in the clinicians' ability to distinguish valid claims from false ones. If our inferences about the underlying truth are wrong, the consequences might be disastrous. As discussed earlier, RCTs are the most commonly used and important tools to address therapeutic issues. When evaluating medical literature for clinical application, the study should be assessed using three discrete steps.^5^

### (i) Are the results valid?

This issue deals with the credibility of the study and whether the results represent an unbiased estimate of the treatment effect. This depends on whether the study was designed and conducted in a way that justifies claims about the therapeutic benefits or harms of a treatment regimen. The validity of the study can be evaluated by finding out whether the persons exposed to a particular intervention or control had similar prognosis at the beginning of the study and whether the groups were still similar with respect to prognostic factors throughout the study.

*(a) Were subjects randomized?* Comparison of outcomes among nonrandomized cohorts of patients who, for various reasons, did or didn't undergo an intervention frequently leads to false conclusions and randomized trials in the past have generated surprises by contradicting the results of these less rigorous trials.^6^–^8^ For example, randomized trials demonstrated that steroid injections do not ameliorate facet-joint back pain^7^ and plasmapheresis does not benefit patients with polymyositis.^8^ The studies in which patient or physician preference determines whether a patient receives treatment or control (or another treatment) often yield biased outcomes. This is because the outcome of interest (like morbidity or mortality) is influenced by many causes of which treatment is only one. The patient's age, severity of the disease condition, dietary factors, lifestyle, presence of co-morbid conditions and a number of other known and unknown factors influence the frequency with which a trial's target outcome occurs. If these factors prove unbalanced between two groups, the outcome will be biased, either overestimating or underestimating the treatment effect. Typically, observational studies are known to overestimate (and occasionally underestimate) treatment effect in comparison to randomized controlled trials.^9^,^10^ Though attempts are made to match patients in observational studies, the power of randomization in balancing the two groups is far greater with respect to both the known and the unknown determinants of outcome.

*(b) Was randomization concealed?* If those determining the patient allocation to a treatment or control group are aware of the arm to which a patient will be allocated, they may knowingly or unknowingly enroll sicker or less sick patients to either the treatment or control group.^11^,^12^ This behavior defeats the purpose of randomization and is likely to yield a biased result. The effective methods of randomization concealment are pharmacy coding of drugs and central concealment in which the recruiter makes a call to a center to find out the arm of allocation before assigning each subject to a particular intervention group.

*(c) Were subjects analyzed in the groups to which they were randomized?* Omitting results of subjects who did not take the assigned treatment or who took alternative treatment will bias the results and ruin the process of randomization. The reason for this is that such behaviors are often shown by patients who are different from the rest of the group in respect to the prognostic factors such as disease severity.^13^,^14^ For example, in comparison of medical or surgical therapy for a particular condition, some patients randomized to surgery never undergo the operation because they are too sick or because they suffer the outcome of interest (e.g., stroke or myocardial infarction) before they are operated. If such patients who otherwise had poor prognosis (as they were sicker) are included in the control arm but excluded from the surgical arm, even a useless surgical therapy will appear to be effective! In reality this apparent benefit has not come from surgery but from the systematic exclusion from the surgical group of those with the poorest prognosis. This process of analyzing all patients in the same group to which they were originally randomized (and not based on the treatment they actually received) is called an intention-to-treat analysis. This process ensures that the known and unknown prognostic factors are equally distributed in two groups and the effect seen is actually the result of the treatment assigned.

*(d) Were patients in two intervention groups similar with respect to known prognostic factors?* Although the process of randomization should take care of this factor, sometimes by chance more patients with a particular risk factor might get allocated to one group. The smaller the trial, more is the chance that prognostic imbalance might be created. Therefore, we should check whether the study subjects in the two groups were similar in respect to at least the known prognostic factors before the commencement of intervention. If the differences are large, the validity of the results is compromised unless statistical measures have been employed to permit adjustment for the baseline differences.

*(e) Was blinding done?* Patients who know that they are taking a treatment they believe to be effective may feel, perform or report better even if there is no biological action. The impact of this placebo-effect or reporting bias on study results is best minimized by ensuring that patients are unaware of the nature of the treatment received and the control group patients benefit from these effects to the same extent as actively treated patients.

Similarly, if the clinicians or/and outcome assessors are aware of the nature of the treatment received, they might prognostically imbalance the groups after randomization by conscious or unconscious differential administration of co-interventions and by differentially interpreting the outcomes, respectively.^15^ Effective blinding eliminates these possibilities and improves the validity of the study results.

*(f) Was follow-up complete?* The subjects which are lost to follow-up often have a different prognosis than those who continue as the reason they are lost may be that they are suffering from adverse effects or on the other hand they might be doing well and so did not return to get assessed. The greater the number of patients getting lost to follow-up, the more is a study's validity potentially compromised.^16^ How much follow-up loss is acceptable depends on the study results. Strict cutoffs like 10% or 20% are often misleading. In assessing whether the study results would have been the same if there was no loss to follow-up, we first assume worst case scenario. This means assuming that all patients allocated to the treatment arm and lost to follow-up suffered from undesired outcome (like death or continuing pain) and all patients allocated to the control group and lost to follow-up had the desired outcome (like survival or pain-free). If assuming this worst case scenario does not alter the magnitude of the treatment effect, the results are valid even with loss to follow-up. If however, this substantially alters the results you have to judge whether the patients lost to follow-up were similar with respect to important prognostic factors such as disease severity. This lessens but does not eliminate the possibility of different rate of target outcome in those who are lost.

### (ii) What are the results?

This involves the interpretation of the size and precision of the study results; and whether and how the results could be applied to the desired clinical setting.^17^

*(a) How large is the treatment effect?* Authors often report the magnitude of benefit in several ways. If in a controlled trial, 5% of those in the treatment group and 10% in control group suffer from an adverse outcome (say death), the absolute risk reduction or risk difference between the proportion who died in the control group and the proportion who died in the treatment group is 0.10-0.05 = 0.05 or 5%. Another commonly reported measure of treatment effect is relative risk reduction which measures the percentage by which the treatment reduces the risk of event relative to that occurring among control patients. In this example, the treatment reduced relative risk of death by (0.1- 0.05)/0.10, i.e., 0.5 or 50%. Whenever the authors report that a certain treatment is x% more effective, we need to examine what the authors mean. Whether they mean absolute risk difference or relative risk reduction has a great bearing on interpretation as often relative risk reduction results in subjective impression of a larger treatment effect.

*(b) How precise is the estimate of treatment effect?* In the above example we have assumed an absolute value of the treatment effect calculated from the observations which is called a point estimate. However, this estimate is unlikely to be precisely correct as the true value lies somewhere in its neighborhood. This is calculated by confidence interval (CI) which is a range of values within which one can be confident that a parameter value lies.^18^ A figure of 95% CI is arbitrarily used which means the range in which the true relative risk reduction value will lie 95% of the times. If the lower value of the 95% CI of the RRR does not have clinical significance, then the trial has not really helped us to decide whether to offer the new treatment or not. In fact, the results could be consistent with an adverse effect of the treatment if the lower value of the 95% CI is in negative despite the point estimate being very high (50% in the above example). In general, smaller the number of patients enrolled in a trial, the wider is the 95% CI and less precise are the results.

### (iii) How can results be applied to patient-care?

The final task in EBM is the process of applying the results of the literature to the clinical practice.^19^,^20^ This involves a detailed knowledge of the clinical setting, patient profile and the issues related to cost and harm.

*(a) Are patients in my practice similar to those in the study?* The profile of patients in our clinical settings might be different from those included in studies. These patients might be more or less sick or suffering from a co-morbid condition like malnutrition or obesity. If our patients would have met the inclusion criteria of the study in question, the results of the study can be safely applied to them. If however, the patients differ in some aspect, we should examine carefully if there is a compelling reason why the results should not be applied to them. If a compelling reason is not found, it is usually safe to generalize the results to your patients.

*(b) Were all clinically important outcomes considered?* The reason we offer treatment to the patients is that it will improve outcomes important for them like reducing symptoms like pain or disability, reducing long-term complications, avoiding hospitalization or preventing death. Many a times, the studies report benefit of treatment in terms of nonfunctional outcomes, for example, improved results of bone densitometry as a result of hormone replacement therapy in osteoporesis or improvement in lipid profile as a result of treatment with a lipid-lowering agent. These surrogate outcomes should not be relied on totally unless the benefit on patient function, comfort or survival is documented along with. Even if the favorable effects of treatment on clinically important outcomes are documented, any deleterious effects on other outcomes need to be examined.

*(c) Are the likely benefits worth the potential harm and cost?* It needs to be seen whether the potential treatment benefits are worth the efforts put by the health system and the patient. A 50% reduction in the relative risk of death may sound very impressive but may be of very minimal impact on the patient or your practice, particularly if the risk of death otherwise is very low. This is illustrated by a term ‘*Numbers needed to treat*’ (NNT) which denotes the number of patients who must receive an intervention to prevent one adverse outcome or produce one positive outcome.^21^ To calculate this, one needs to know the risk of the adverse event if left untreated. Suppose the risk of death due to a disease condition is 1 in 500 (0.2% or 0.002). If the intervention reduces the risk of death by 50% (RRR of 0.5), the risk of death after treatment becomes 0.1% or 1 in 1000. The absolute risk reduction (ARR) due to this treatment strategy becomes 0.2-0.1% = 0.1% or 0.001. The inverse of this ARR is equal to the number of such patients we would have to treat to prevent one event-the NNT. In this example, we need to treat 1000 patients to save a single life. If the drug otherwise is toxic, the number harmed might become more than the lives saved. On the other hand, if the risk of death due to a particular condition is high, the ARR would become larger with same RRR and NNT would be smaller making the drug more useful. Knowing the NNT helps clinicians in the process of trading off the benefits and risks associated with the management options.

## BEYOND THERAPEUTICS

In the foregoing, we have dealt mainly with therapeutic issues particularly in Step 3 and Step 4. Application of EBM is equally important to other issues like those of harm (adverse events of therapy), diagnosis^22^ and prognosis.^23^ The process of evaluating evidence and clinical application for these is almost similar, though we need to examine different kind of studies, say observational studies for issues related to harm and sensitivity/specificity studies for performance of a diagnostic test. Similarly, systematic reviews need to be examined for their validity of results and clinical applicability.^24^ Thorough description of these is beyond the scope of this article and readers are encouraged to consult detailed texts and reviews on these topics.^22^–^30^

## LIMITATIONS OF EBM

The examination of the concepts and practice of EBM by clinicians and academicians has led to negative as well as positive reactions. The need to develop new skills in searching and critical appraisal of the literature can be daunting. Second, busy clinicians have limited time to master and apply these new skills and the resources required for instant access to evidence are often woefully inadequate in clinical settings. Providing evidence-based care directed toward maximizing patients' quality of life often increases the costs of their care. Also, evidence that EBM “works” has been late and slow to come.

However, the earlier sections have clarified some “pseudolimitations” that arise from misunderstandings of the definition of EBM. An examination of the definition and steps of EBM quickly dismisses the criticisms that it denigrates clinical expertise, is limited to clinical research, ignores patients' values and preferences or promotes a cookbook approach to medicine. In fact, it involves all these issues in a more systematic and extensive manner.

### Obstacles to EBM

It is feared that nonimplementation or partial implementation is likely to be the fate of many interventions based on EBM.^31^ There are several behavioral obstacles to implementation of evidence in practice.^32^ Firstly, much of the science is seen in practice as inconclusive or as contested. Secondly, it is because groups of professionals retain substantial autonomy over their work practices and resist external interventions from a generally marginal and powerless research and development function. Thirdly, it is because much clinical knowledge is tacit and experiential in nature and thus seen as more of a craft than a science, so that the findings of EBM are not fully accepted by practitioners as valid in their practice.

Several other important nonscientific influences over decision-making are also obstacles to the practice of EBM. These include the fear of possible medico-legal intervention; the need to ensure ease of administration of the drug across the clinical group; the successful marketing of a new drug by drug companies; imitative behavior as a critical mass of colleagues adopts a new modality; and unhappy experience with individual patients which creates a counter reaction.^31^

The solution most probably lies in self-regulation on the part of clinicians and pharmaceutical companies. Though there is a hard felt need of behavioral change in practice, this has to be led by the professional groupings themselves and cannot be imposed from outside. Professional bodies should demonstrate that they are taking an active role in self-regulation.
